# Host kinin B1 receptor plays a protective role against melanoma progression

**DOI:** 10.1038/srep22078

**Published:** 2016-02-22

**Authors:** Andrea G. Maria, Patrícia Dillenburg-Pilla, Rosana I. Reis, Elaine M. Floriano, Cristiane Tefé-Silva, Simone G. Ramos, João B. Pesquero, Clara Nahmias, Claudio M. Costa-Neto

**Affiliations:** 1 Department of Biochemistry and Immunology; Ribeirão Preto Medical School – University of São Paulo, 14049-900, Ribeirão Preto, Brazil; 2 Departament of Pathology, Ribeirão Preto Medical School – University of São Paulo, 14049-900, Ribeirão Preto, Brazil; 3 Department of Biophysics, Federal University of São Paulo, 04039-032, São Paulo, Brazil; 4 Inserm U981,Institut Gustave Roussy, 94800, Villejuif, France

## Abstract

Melanoma is a very aggressive tumor that arises from melanocytes. Late stage and widely spread diseases do not respond to standard therapeutic approaches. The kallikrein-kinin system (KKS) participates in biological processes such as vasodilatation, pain and inflammatory response. However, the role of KKS in tumor formation and progression is not completely understood. The role of the host kinin B1 receptor in melanoma development was evaluated using a syngeneic melanoma model. Primary tumors and metastasis were respectively induced by injecting B16F10 melanoma cells, which are derived from C57BL/6 mice, subcutaneously or in the tail vein in wild type C57BL/6 and B1 receptor knockout mice (B1^−/−^). Tumors developed in B1^−/−^ mice presented unfavorable prognostic factors such as increased incidence of ulceration, higher levels of IL-10, higher activation of proliferative pathways such as ERK1/2 and Akt, and increased mitotic index. Furthermore, in the metastasis model, B1^−/−^ mice developed larger metastatic colonies in the lung and lower CD8^+^immune effector cells when compared with WT animals. Altogether, our results provide evidences that B1^−/−^ animals developed primary tumors with multiple features associated with poor prognosis and unfavorable metastatic onset, indicating that the B1 receptor may contribute to improve the host response against melanoma progression.

Melanoma is a very aggressive tumor that arises from melanocytes; a cell type specialized in producing the skin protective pigment melanin. Although early stage-diagnosed disease is highly curable by surgical removal of the malignancy, late stage and widely spread diseases do not respond to standard therapeutic approaches, such as chemotherapies and radiotherapies. Consequently, the survival rate of patients drops from 90% in early stages to only 10% in late-stage melanoma, with a recurrence risk of up to 60% in the last case^1^. Activating mutations in proto-oncogenes or inactivating mutations in tumor suppressor genes are major players in tumor formation, and indeed, BRAF mutations have been shown to be highly prevalent among melanomas^2^,^3^. Although the blockade of this pathway improved patient outcome, many cases of tumor relapse were reported^4^. Furthermore, in many cases, added to mutations, tumors require a permissive microenvironment to confer a complete malignant and invasive phenotype^5^,^6^. The ability to change its microenvironment and hijack host pro-inflammatory and migratory signaling capacity is crucial to promote tumor sustained proliferative signals, induce angiogenesis and promote tumor-related inflammation. This dynamic interface between host and tumor cells is still poorly understood, however it has been shown to actively modulate tumor aggressiveness^7^.

The kallikrein-kinin system (KKS) is responsible for several biological processes, such as vasodilatation/vasoconstriction, modulation of pain, inflammatory response, contraction/relaxation of smooth muscles and cell proliferation^8^. The two main receptors of the KKS are kinin B1 and kinin B2 receptors. While the kinin B2 receptor is constitutively expressed under physiological conditions and binds with high affinity to bradykinin (BK) and kallidin (KD), the expression of the kinin B1 receptor is inducible by a range of factors that include exposure to agonists, such as des-Arg^9^-BK (DABK) or des-Arg^10^-KD (DAKD), pathological conditions, inflammation, activation of kinin B2 receptor, as well as other factors that are able to engage and activate the transcription factors CREB, AP1 and NFκB^9^,^10^,^11^.

Regarding tumor formation and progression, the role of KKS in this pathology is still poorly understood. The expression of kinin B1 and B2 receptors has been described in many tumor types, and several reports have shown a pro-tumor role of kinin B2 receptor^12^,^13^,^14^,^15^. For the kinin B1 receptor, a much less clear scenario appears. Although its activation has been related to induction of cell proliferation and primary tumor growth in lung and prostate cancer xenografts^16^, a recent report from our group showed that activation of kinin B1 receptor in tumor cells reduces melanoma progression, significantly decreasing metastasis and therefore improving animal survival^17^. Regarding the contribution of the B1 receptor present in the host, there is no data in the literature describing its role during melanoma development and metastasis. Considering that the tumor microenvironment has been shown to play a major role in tumor growth and progression and that kinin B1 receptor activation in tumor decreased tumor burden and extended animal life span, we hypothesized that kinin B1 receptor in the tumor microenvironment could contribute to a host protective response against melanoma.

In the present work we investigated whether kinin B1 receptor present in the tumor microenvironment could also contribute to the host response during melanoma progression. To that, we took advantage of B16F10 melanoma cells, which is a well validated model for both primary tumor and pulmonary metastasis^18^,^19^,^20^. Using the kinin B1 knockout mice (B1^−/−^) and a syngeneic tumor model, we show that the absence of the host kinin B1 receptor gives rise to melanomas with higher incidence of ulceration, decreased immune response, higher mitotic index and larger metastatic colonies, all of them markers of poor prognosis^21^,^22^.

## Results

### Ablation of the kinin B1 receptor in mice leads to a higher incidence of ulcerated tumors

**Figure 1:**
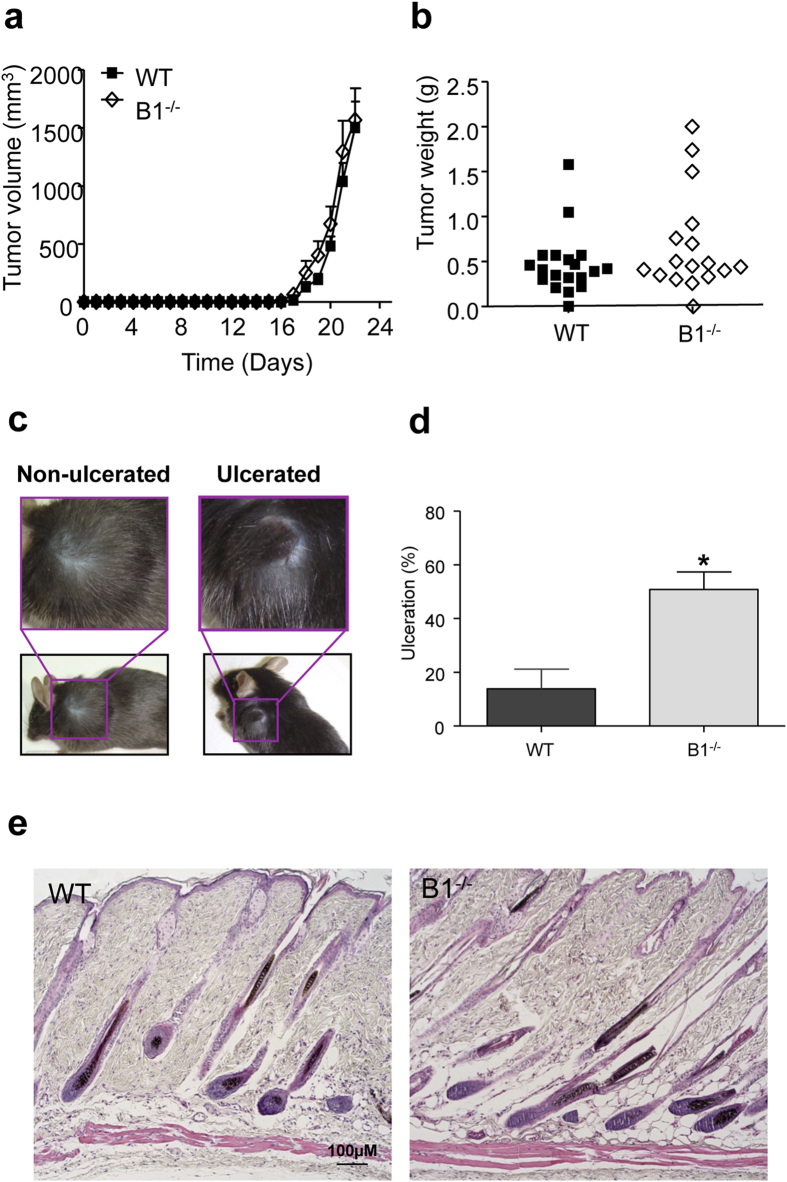
Ablation of the host kinin B1 receptor increases skin ulceration. (**a**) Tumor growth curve of wild type (WT) and kinin B1 receptor-knockout mice (B1^−/−^) after receiving subcutaneous injection of 300,000 B16F10 cells. (**b**) Tumor weight 22 days after B16F10 cells inoculation; WT: n = 19, B1^−/−^: n = 17, p = 0.1758. (**c**) Representative images of C57BL/6 animals with non-ulcerated and ulcerated B16F10 melanomas. (**d**) Percentage of animals that showed a breakdown of the skin over the melanoma (ulcerated lesions) 22 days after receiving subcutaneous injection of 300,000 B16F10 cells. Data are expressed as a percentage ± SEM; WT: n = 19, B1^−/−^: n = 17, *p = 0.0197. (**e**) Representative images from histological analysis of skin of adult mice showing no changes in skin architecture between wild type (WT) and kinin B1 receptor-knockout mice (B1^−/−^); n = 6.

To evaluate the contribution of the host kinin B1 receptor in the tumor microenvironment for melanoma growth, we performed a melanoma implantation assay by inoculating 300,000 B16F10 cells into the dorsal superior region of WT and B1^−/−^ mice. Tumor growth was monitored daily. Results show that in both groups, tumors remained quiescent for a long period of time before growing extremely rapidly during the last 5 days of the experiment to reach maximal volume at day 22 (Fig. 1a). As we observed very similar kinetic profiles of tumor growth for both animal groups, and considering that kinin B1 receptor is induced under pathological conditions, we performed the analyses at the end point of the tumor growth curve, which would allow more time for host response and kinin B1 receptor expression to be induced in the WT mice and subsequent engagement in the possible downstream responses and outcomes. Despite that the kinetics of tumor progression (Fig. 1a) and tumor weight at the experiment end point (Fig. 1b) were not significantly altered in B1-deficient mice, significant differences between tumors from WT and B1^−/−^ mice were found regarding one of the key prognostic markers for melanomas, which is ulceration. The presence of ulceration in the primary tumor is a strong indicator of poor prognosis and it is considered one of the most powerful survival predictor for melanoma patients, along with tumor thickness and mitotic rate^21^,^22^. According to the American Joint Committee on Cancer (AJCC), since 2009 the presence of ulceration has to be considered for the T-classification of primary melanomas. Non-ulcerated tumors are classified as Ta while ulcerated tumors are classified as Tb. Here, we considered ulceration when a disrupted epidermal continuity was observed around the tumor area^23^, as shown in Fig. 1c. Approximately 50% of the tumors that developed in the B1^−/−^ mice were ulcerated, compared to only 10% of the tumors in the WT animals (Fig. 1d), although there was no difference in the size of the ulcerated lesions. It is important to note that histological analysis of skin from kinin B1^−/−^ mice revealed no alteration in the skin architecture when compared with WT animals (Fig. 1e). These results suggest that tumors developing in a B1-deficient microenvironment may show similar growth rate, but increased aggressiveness as compared to tumors developing in WT mice. Taken together, melanomas from kinin B1 receptor deficient mice could be classified in a different stage group such as more aggressive melanomas, suggesting that the presence of the B1 receptor in the host may contribute to a protective response against melanoma.

### Ablation of the kinin B1 receptor in mice does not affect melanoma vascularization

**Figure 2:**
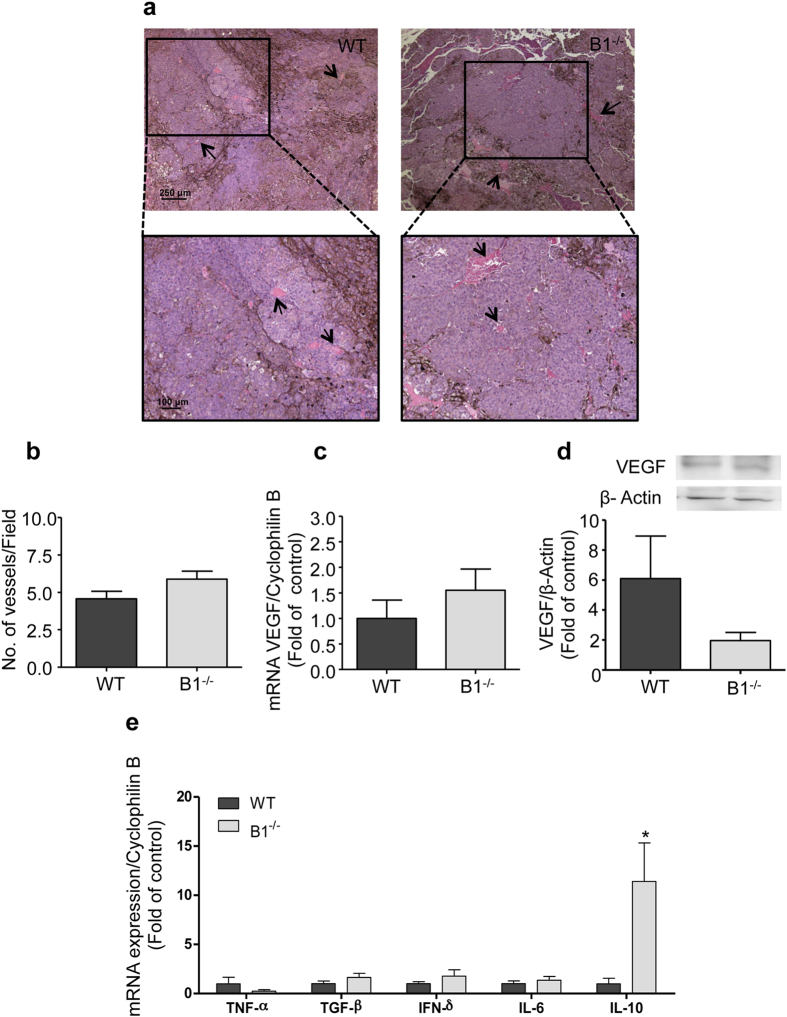
Ablation of the host kinin B1 receptor does not alter melanoma vascularization but presents increased IL-10 levels. **(a**) Representative images from histological analysis showing similar vascular network (black arrows) of tumors developed in wild type (WT) and kinin B1 receptor-knockout mice (B1^−/−^) 22 days after melanoma cells inoculation. **(b)** Quantification of the number of vessels in tumors from WT and B1^−/−^ mice in ten different high magnification fields. Data are expressed as number of vessels ± SEM; n = 8, p = 0.1127. **(c)** Quantitative real-time PCR analysis of VEGF mRNA expression in B16F10 tumors from WT and B1^−/−^ mice. Data are expressed as fold of change from relative expression of VEGF/Cyclophilin B ± SEM; n = 12, p = 0.3246. **(d)** VEGF expression was accessed by western blotting 22 days after tumor cells implantation in wild type (WT) and B1 receptor-knockout mice (B1^−/−^). Data are expressed as fold of change from relative expression of the VEGF/β-actin ± SEM; n = 5; p = 0.1074. **(e)** Analysis of cytokine profile in B16F10 tumors from wild type (WT) and kinin B1 receptor- knockout mice (B1^−/−^) 22 days after subcutaneous injection of melanoma cells. Experiments were performed by quantitative real-time PCR. Data are expressed as fold of change from relative expression of target gene/Cyclophilin B ± SEM; n = 12, *p = 0.0107.

Angiogenesis is a crucial process during tumor formation and therefore is considered one of the hallmarks in cancer. Although it is not a direct prognostic marker in melanomas, the ability to build a vascular network allows glucose and oxygen supply to the growing tumor^24^. We compared tumor vascularization between melanomas from WT and B1^−/−^ mice. Representative images from histological analysis of the tumors and quantification of the number of vessels per high magnification field showed no significant difference in the tumor vascularization between both groups (Fig. 2a,b). As VEGF is a key player in angiogenesis inducing endothelial cells proliferation^25^ as well as vascular permeability^26^ we analyzed VEGF mRNA expression by real-time PCR and protein expression by western blotting in tumors from WT and B1^−/−^ mice. As seen in Fig. 2c,d, both groups express similar levels of VEGF, suggesting that host kinin B1 receptor contributes neither to neovascularization nor to VEGF-dependent vascular permeability during melanoma development.

### Kinin B1 receptor-knockout mice show tumors with increased IL-10 levels

As high immune response has been correlated with good prognosis in melanomas^27^ and activation of B1 receptor in tumor cells has been shown to decrease melanoma development^17^, we hypothesized that absence of host kinin B1 receptor could potentially impact cytokine production in the tumor area, which ultimately would lead to altered host immune response. Therefore, we evaluated cytokine expression profiles in tumors from WT and B1^−/−^ mice. As seen in Fig. 2e, although there are no significant changes in the expression of pro-inflammatory cytokines, such as TNF-α, TGF-β, INF-γ and IL-6, we found a significant overexpression of the anti-inflammatory cytokine IL-10 within the tumor mass of B1^−/−^ mice. This result suggest that in the absence of host kinin B1 receptor, tumors grow in an IL-10 enriched microenvironment, which possibly contributes to down-regulate host immune response^28^.

### Ablation of the kinin B1 receptor in mice increases activation of proliferative pathways and mitotic index in melanoma

**Figure 3:**
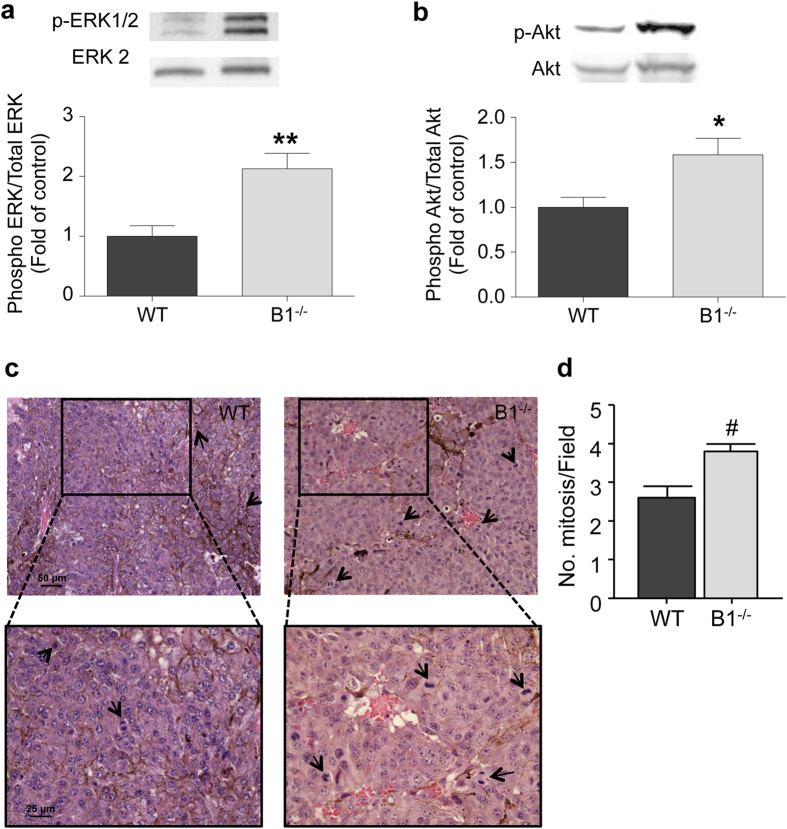
Ablation of the host kinin B1 receptor increases activation of proliferative pathways and mitotic index in melanoma. Activation of proliferative pathway was accessed by western blotting 22 days after tumor cells implantation in wild type (WT) and B1 receptor-knockout mice (B1^−/−^). **(a)** Phosphorylation/activation of ERK and **(b)** AKT, are increased in tumors from B1^−/−^ mice. Data are expressed as fold of change from relative expression of the phosphorylated/total form of the protein ± SEM; n = 8; **p = 0.0028, *p = 0.0120. **(c)** Representative images from histological analysis showing a higher number of cells in mitosis (black arrows) in tumors from B1^−/−^. **(d)** Quantification of the number of cells in mitosis in tumors from WT and B1^−/−^ mice in ten different high magnification fields. Data are expressed as number of cells ± SEM; n = 8; ^#^p = 0.0098.

Among different prognostic factors used to classify tumor aggressiveness, the mitotic index is the most widely used and accepted in the clinic^29^. To gain further insights regarding the role of host kinin B1 receptor in melanoma tumor growth and progression, we analyzed activation of proliferative pathways. As seen in Fig. 3a, ERK1/2 phosphorylation is increased in tumors from B1^−/−^ mice. Moreover, PI3K-Akt pathway, another important survival/proliferative pathway was also significantly more activated in tumors from B1^−/−^ as evaluated from Akt phosphorylation at Ser^308^ (Fig. 3b). Histological analysis of the tumors from WT and B1^−/−^ mice support our molecular analysis and show a higher incidence of cells undergoing mitosis in tumors from B1^−/−^ mice when compared to WT (Fig. 3c). To calculate the mitotic index of tumors from WT and B1^−/−^ mice, a pathologist analyzed tumor samples from WT and B1^−/−^ mice and counted the number of cells in mitosis in 10 non-coincident high magnification fields. Histopathological analysis showed a significant higher number of cells undergoing mitosis in tumors from B1^−/−^ mice (Fig. 3d), confirming the more aggressive feature of tumors developed in the absence of kinin B1 receptor.

### Melanoma metastases analysis in kinin B1 receptor-knockout mice shows larger colonies in the lung, with increased vascularization and decreased infiltration of effector CD8^+^T cells

**Figure 4:**
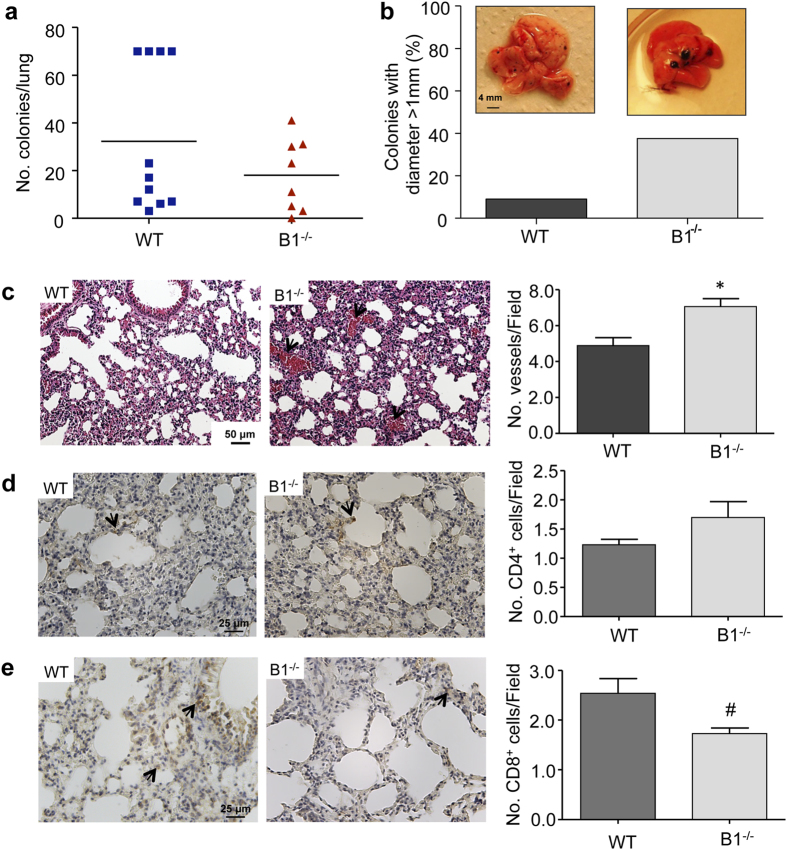
Kinin B1 receptor in the host in involved in colony growth and immune response during metastasis progression. Lung metastasis was induced by the injection of B16F10 cells in the tail vein of wild type (WT) and kinin B1 receptor-knockout mice (B1^−/−^) WT: n = 11, B1^−/−^: n = 8. **(a)** Number of colonies stablished in the lung after 30 days of B16F10 cells injection; p = 0.2416. **(b)** Percentage of colonies with diameter higher than 1 mm. **(c)** Histological analysis showing higher number of blood vessels (black arrows) in metastatic lungs of B1^−/−^ compared to WT mice. Data are expressed as number of vessels ± SEM; n = 8, *p = 0.0036. **(d)** Immunohistochemistry analysis of CD4^+^and **(e)** CD8^+^cells in the metastatic lung from WT and B1^−/−^ mice. Data are expressed as positive cells ± SEM ^#^p = 0.0132; n = 6. **(c–e)** Left panel: representative images, right panel: quantification of ten different high magnification fields.

To study whether host kinin B1 receptor could play a protective role in metastatic melanoma, B16F10 melanoma cells were delivered directly into the blood flow of WT and B1^−/−^ mice by tail vein injection, and within 30 days after injection lung metastases were screened. As shown in Fig. 4a, there was no significant difference between the number of colonies present in the lungs of WT and B1^−/−^ mice. However, the incidence of large melanoma colonies in the lungs was 3 fold higher in B1^−/−^ mice compared with the WT group (Fig. 4b). Corroborating this data, histological analysis revealed a significant increase in the number of blood vessels in the lungs from B1^−/−^ mice (Fig. 4c), which suggests that absence of host kinin B1 receptor confers growth advantages to melanoma in the lung. We performed full autopsies and as expected for this model^30^, we only found metastasis in the lungs with no incidence of tumors spread into other organs. Given that host immune response is a key component during tumor growth and progression^31^, that high immune response has been correlated with better prognosis in melanomas^27^, and that our primary tumor data showed a remarkable increase in tumor expression of the anti-inflammatory cytokine IL-10 when host B1 receptor is absent (Fig. 2e), we investigated whether the content of immune cells in metastatic lungs of WT and B1^−/−^ mice was altered. Using immunohistochemistry assay, we analyzed the content of CD4^+^T cells as well as of the effector CD8^+^T cells in the metastatic lungs of WT and B1^−/−^ mice. Interestingly, we found effector CD8^+^T cells to be significantly reduced in the lungs of B1^−/−^ mice (Fig. 4e). In contrast we found that both groups presented similar levels of CD4^+^T cells in the lungs (Fig. 4d), which suggests that absence of host kinin B1 receptor did not impair CD4^+^T cell migration to the metastatic lung but increases CD8^+^cells. Moreover, we assessed IL-10 expression levels in the lungs, but no significant differences were found when comparing wild type and B1^−/−^ mice (Supplementary Figure S1). Taken together our data show that loss of host kinin B1 receptor affects two major cancer-related events in metastatic melanomas: tumor cell proliferation and host anti-tumor immune response. Therefore, our data suggest that host kinin B1 receptor may play a role against tumor progression during melanoma metastasis formation.

## Discussion

The expression of KKS components has been reported in several malignancies. For instance, PSA (prostate specific antigen), a widely used diagnostic marker for prostate tumors^32^, is a kallikrein. Recent studies have started uncovering the underling mechanisms by which the KKS is involved in tumorigenesis; however, the role of kinin receptors in this process is not fully understood. The KKS is directly affected by class I anti-hypertensive drugs, since the blockade of ACE/kininase II promotes accumulation of kinin receptors ligands. Considering the widespread usage of ACE inhibitors in the treatment of cardiovascular and renal diseases^33^, understanding the role of kinin receptors in tumorigenesis may lead to better therapeutic approaches. Although, there are indications that the antagonism of the kinin B1 receptor decreases cell proliferation in different tumor cells^12^, nothing can be found in the literature regarding the role of the kinin B1 receptor present in the host during tumor development. Here, we bring evidence that the host kinin B1 receptor may contribute towards a protective role against melanoma progression. Using a syngeneic melanoma model, we induced melanoma in WT and B1^−/−^ mice and observed that B1^−/−^ mice tumors showed higher levels of the anti-inflammatory cytokine IL-10, increased incidence of ulceration as well as increased mitotic index; all features that are considered as markers of poorer prognosis for melanoma patients^21^. Moreover, using an experimental metastasis model, we showed that B^−/−^ mice developed larger lung metastatic colonies, increased lung vascularization, and decreased infiltration of anti-tumor CD8^+^T cells as compared to WT animals.

The KKS and its vasoactive peptides BK, desArg^9^-BK, KD and desArg^10^-KD play important roles in vasodilation, smooth muscle contraction, and inflammation. Although the connection between inflammation and cancer was proposed in the 19^th^ century by Rudolph Virchow^34^, and has recently been considered a hallmark of cancer^6^, the role played by immune cells within the tumor area is still not fully understood. While the presence of inflammatory cells has been associated with poor prognosis in many solid tumors^35^, they are also essential for host anti-tumor immune response. Our data show that in the absence of the host kinin B1 receptor, the expression of pro-inflammatory cytokines such as TNF-α, IFN-γ, IL-6 and TGF-β are unchanged in primary tumors. However, we observed that the anti-inflammatory cytokine IL-10 was upregulated, promoting an IL-10 preponderant microenvironment within the tumor mass. Considering that activation of B1 receptor triggers pro-inflammatory responses^36^,^37^, we believe that the lower levels of IL-10 in primary tumors from WT animals might be due to the pro-inflammatory action of the WT host B1 receptor, which in this case can counteract the anti-inflammatory response within the tumor. Regarding metastasis, we did not find significant differences of IL-10 expression in lungs with metastatic melanoma colonies from WT and B1^−/−^ animals. It is however important to note that, although the majority of cells from primary tumor samples are comprised of tumor derived cells, small tumor colonies from lungs are processed with a larger area of host lung parenchyma. In fact, local IL-10 affects host immune response in many levels, including decrease in CD8^+^T-cell-mediated tumor lysis, down regulation of major histocompatibility complex (MHC) class II and co-stimulatory molecules in dendritic cells^38^,^39^. Moreover, the kinin B1 receptor has been already associated with the migration of immune cells to injured tissues^40^. In our study, we observed that lungs from WT animals presented lower number of vessels compared to B1 receptor deficient mice. The less vascularized microenvironment along with increased CD8^+^T-cell infiltration in the lungs, may have at least partially contributed to arrest colony growth in the lungs, resulting in the smaller colony size phenotype observed in WT animals.

The AJCC serves as the dominant staging system for melanomas, which is pivotal in determining prognosis and appropriate treatment. For primary tumor evaluation, since 2009 the AJCC takes into account ulceration and mitotic activity for the pathological assessment; and the presence of both parameters is directly correlated with poorer prognosis^41^,^42^,^43^. Our data show that in the absence of the host kinin B1 receptor the incidence of ulcerated tumors increased from 10% in WT to 50% in B1^−/−^ mice. BRAF mutations are highly prevalent in melanomas, suggesting that this type of tumor, between other pathways, is regulated by the MAPK pathway. In fact, the use of BRAF inhibitors have been approved to treatment of melanoma and, although recurrence has been reported, most of the cases are associated with the plasticity of the tumors to reactivate MAPK or upregulate the alternative pathway, PI3K/Akt^44^. Interestingly, our data show that the absence of host kinin B1 receptor allows up-regulation of MAPK/ERK and PI3K/Akt pathways, which correlates with the increased mitotic index in tumors from B1^−/−^. These results corroborate our hypothesis that the absence of the B1 receptor in the microenvironment may increase tumor anti-inflammatory responses, depressing the immune system and consequently providing better conditions for cell proliferation and tumor aggressiveness. Studies with melanoma patients showed that higher mitotic rate is correlated with ulceration and thicker melanomas^45^. Our data show that B1^−/−^ mice developed tumors with higher mitotic index along with higher ulceration than WT mice, while both groups share similar tumor size. It is well established that multiple factors contribute to the primary tumor size, especially the ratio between tumor cells proliferation and death, which can induce feedback loops modulating growth, angiogenesis, host immune response and ultimately, tumor progression and metastasis. In this context, one possibility is that tumors from host deficient kinin B1 receptor, which presented 25% higher mitosis index than WT (Fig. 3c,d) but similar primary tumor volume (Fig. 1a,b) and similar vascular network density (Fig. 2a,b), could be in shortage of nutrients and oxygen supply, which could ultimately be a limiting factor for primary tumor growth. However, even with a scarcity of nutrients and oxygen supply, the alterations caused by the absence of the kinin B1 receptor in the lung microenvironment would be sufficient to modulate tumor-host interaction and favor metastasis. Indeed, we found that lung colonization by melanoma cells in the metastatic model presented larger colonies, suggesting that despite of similar primary tumors volume; kinin B1 receptor deficient mice give rise to more aggressive and metastatic tumors than WT mice.

Several reports have shown expression of kinin receptor in tumors and tumor cell lines^14^,^15^,^46^, and the use of antagonists, mainly for the B2 receptor, has been shown to decrease tumor growth and proliferation in many tumor types^12^,^13^, suggesting a pro-tumor role for the kinin B2 receptor. A less clear scenario is observed regarding the kinin B1 receptor. Although many reports showed that the B1 receptor contributes to tumor growth and progression, most of the studies have been done in systems that co-express B1 and B2 receptors. In fact, a cross-talk between those receptors has been described^47^, suggesting that a pro-tumor role of the B1 receptor might depend on B2 receptor expression. A recent report from our group using a B2-free cellular system showed that activation of kinin B1 receptor in the tumor cells resulted in less aggressive tumors, as evidenced by decreased proliferation, vascularization and metastasis, which ultimately improved animal survival^17^. Here, using a syngeneic model in a B1 receptor free microenvironment, we show that targeting the kinin B1 receptor from the host resulted in tumors with unfavorable prognostic factors. Therefore, we believe that the host kinin B1 receptor may contribute to improve the host response against melanoma progression. Considering that metastatic melanoma still represents a great challenge in terms of aggressiveness and therapy resistance, targeting kinin B1 receptor may open new perspectives to improve the treatment of this disease.

## Materials and Methods

### Animal care and Ethics statement

All methods were performed in accordance with the guidelines of the Brazilian College of Animal Experimentation (COBEA), and all experimental protocols were approved by the Commission of Ethics in Animal Research (CETEA) from the Ribeirao Preto Medical School, University of São Paulo (CETEA, protocol 003/2011).

The B1 receptor-knockout mice (B1^−/−^) and the control littermates in a C57BL/6 genetic background were obtained from the Department of Biophysics, Federal University of São Paulo. The mice were bred and housed in a specific pathogen-free facility with the room temperature controlled at 25 °C in a 12 h light/dark cycle; mice received food and water *ad libitum*. Euthanasia was conducted by cervical dislocation at the study endpoint or earlier if animals met any of early removal criteria (lethargy hunched posture or ruffled coat).

### Reagents

DNAse and SYBR Green reagents were purchased from Invitrogen (Carlsbad, CA, USA). The Improm-II reverse transcription system was purchased from Promega (Madison, WI, USA), and primers were purchased from IDT. Primary antibodies were purchased either from Millipore (Billerica, MA, USA), Cell Signaling Technology (Beverly, MA, USA), Abcam (Cambridge, United Kingdon) or Santa Cruz Biotechnology (Dallas, TX, USA); horseradish peroxidase-conjugated secondary antibodies were purchased from Millipore (Billerica, MA, USA), and the ECL kit was purchased from GE Healthcare (Buckinghamshire, England). Cell culture media and supplements were purchased from Gibco (Carlsbad, CA, USA).

### Cell culture

B16F10 cells, which are derived from C57BL/6 mice, were cultured in Ham’s F10 media at pH 6.9 supplemented with 10% FBS and 10 μg/mL of gentamicin. All of the experiments were performed with 80–90% confluent cells.

### *In vivo* studies

Primary tumor studies were performed using B16F10 melanoma cells. Tumors were induced injecting 300,000 cells in 100 μL of PBS subcutaneously into the dorsal superior region of WT (n = 19) or B1^−/−^ (n = 17) mice weighing approximately 25 g. Tumor size and animal weight were monitored daily for 22 days. At the study endpoint, animals were euthanized, and tumor samples were collected. Half of the tumor samples were immediately frozen in liquid nitrogen and pulverized for RNA and protein extraction. The remaining tumors were fixed in a 10% formaldehyde solution for histological analysis.

The metastatic model was induced by injecting 250,000 B16F10 melanoma cells in 100 μL of PBS in the tail vein of WT (n = 11) or B1^−/−^ (n = 8) mice weighing approximately 25 g. Animal weight was monitored daily for 30 days. At the study endpoint, the animals were euthanized, and lungs were collected. The samples were fixed in a 10% formaldehyde solution for histological analysis.

### Gene expression analyses

Total RNA was extracted with the TRIzol reagent from 100 mg of tumor sample, following the manufacturer’s instructions (Invitrogen). One microgram of total RNA was used for DNAse treatment and subsequent reverse transcription using the Improm-II protocol. For gene expression analysis, we performed quantitative PCR (qPCR) using 10–50 ng of cDNA and platinum SYBR Green qPCR supermix UDG with ROX reference dye (Invitrogen, Carlsbad, CA); results were analyzed using the ABI Prism 7000 sequence detection system. Transcripts were quantified relative to the housekeeping gene cyclophilin B using the ct method^48^. For gene expression assessment, we analyzed samples from 12 animals from each group, WT and B1^−/−^. The oligonucleotide primers used in the PCR analyses are listed in Supplementary Table S1.

### Western Blotting

Protein extraction from pulverized tissues was performed using the following lysis buffer: 10 mM Tris-HCl, pH 7.5; 150 mM NaCl; 1 mM EDTA; 1 mM EGTA; 0.1% SDS; 1% Nonidet P-40; 1 mM PMSF, 10 μg/mL leupeptin, 100 μg/mL aprotinin, 10 mM benzamidine, 1 mM NaF, 1 mM sodium orthovanadate, and 1 mM DTT. The cell lysates were swirled for 30 minutes at 5 °C and centrifuged at 13500 rpm for 15 minutes (Eppendorf Centrifuge 5417 R). The supernatant was subsequently analyzed for total protein content using the Bradford quantification method (Bio-Rad, Hercules, CA). Fifty micrograms of protein was loaded into 12% acrylamide gels and separated by SDS-PAGE. Proteins were subsequently transferred onto a nitrocellulose membrane, blocked with 1% BSA and incubated with antibodies against VEGF (Abcam), β-actin (Millipore), phospho-ERK1/2 and ERK2 (Santa Cruz Biotechnology), phospho-Akt and Akt (Cell Signaling). Anti-mouse or anti-rabbit horseradish peroxidase-conjugated secondary antibodies and an ECL kit were used to visualize immunoblots. Quantification by densitometry was performed using ImageJ software^49^.

### Histopathological analyses

Tumor samples were collected with adjacent tissue to preserve the tumor microenvironment and fixed in a 10% formaldehyde solution. Paraffin blocks were prepared, sectioned (4 μm) and stained with hematoxylin and eosin. Slides were analyzed using a Leitz Aristoplan microscope (Germany) coupled to a Leica model DFC280 color camera (Heerbrugg, Switzerland). Mitotic cells and vessels from the primary tumor and from the lung were quantified at magnifications of 400x and 200x, respectively, across 10 random, non-coincident microscopy fields. Skin samples were visualized at a magnification of 100x.

### Immunohistochemistry

Formalin-fixed paraffin-embedded samples were sectioned into 4 μm slices. Deparaffinized sections were subjected to antigen retrieval with citrate solution, pH 6.0 and treated with 3% hydrogen peroxide to inhibit endogenous peroxidase activity. After blocking slides with fetal bovine serum, sections were incubated overnight at 4 °C with a 1:100 dilution of CD4 monoclonal antibody (Abcam) and CD8 monoclonal antibody (Santa Cruz Biotechnology). Slides were treated with biotin-conjugated secondary anti-mouse antibody (Dako) and with horseradish peroxidase–conjugated avidin (Dako). Peroxidase activity was localized for all samples with 3,3′-diaminobenzidine, counterstained with Harrys’s hematoxylin, dehydrated, cleared and mounted in mounting medium (Entellan, Merck).

### Image Quantification

For evaluation of the presence CD4^+^and CD8^+^cells, we determined the surface density by optical density in the image analysis using the Leica QWin software (Leica Microsystems Image Solutions, Cambridge, UK) in conjunction with a Leica DMR microscope (Leica, Microsystems GmbH, Wetzlar, Germany), video camera (Leica Microsystems, Heerbrugg, Switzerland) and an online computer. The thresholds for CD4^+^and CD8^+^cells were established for each slide after enhancing the contrast to a point at which the cells were easily identified as a brown staining. Ten randomly chosen, non-coincident fields were measured for each group at 200x across a total area of 2.3 mm^2^.

### Statistical analysis

Statistical analysis was performed using Student’s *t*-tests, two-tailed. Data are expressed as mean ± SEM. Differences between mean values were considered significant when p < 0.05.

## Additional Information

**How to cite this article**: Maria, A. G. *et al.* Host kinin B1 receptor plays a protective role against melanoma progression. *Sci. Rep.* **6**, 22078; doi: 10.1038/srep22078 (2016).

## Supplementary Material

Supplementary Information
