# Supplementary material for: Host kinin B1 receptor plays a protective role against melanoma progression
Source: Sci Rep. 2016 Feb 22;6:22078. doi: 10.1038/srep22078 (PMC4761993; doi:10.1038/srep22078)
Supplement: Supplementary Information [file srep22078-s1.pdf]

Supplementary Material – Maria et al, 2016. Sci. Rep.

Host kinin B1 receptor plays a protective role against melanoma progression

Andrea G. Maria[1], Patrícia Dillenburg-Pilla[1], Rosana I. Reis[1], Elaine M. Floriano[2], Cristiane Tefé-Silva[2], Simone G. Ramos[2], João B. Pesquero[3], Clara Nahmias[4], Claudio M. Costa-Neto[1*].

[1]Department of Biochemistry and Immunology; [2]Departament of Pathology, Ribeirão Preto Medical School – University of São Paulo, 14049-900 – Ribeirão Preto, Brazil; [3]Department of Biophysics, Federal University of São Paulo, 04039-032 – São Paulo, Brazil; [4]Inserm U981, Institut Gustave Roussy, 94800 – Villejuif, France.

*Corresponding author:

Claudio M. Costa-Neto, PhD

Phone: +55 16 3315 3261

Fax: +55 16 3633 6840

E-mail address: claudio@fmrp.usp.br

Supplementary Figure

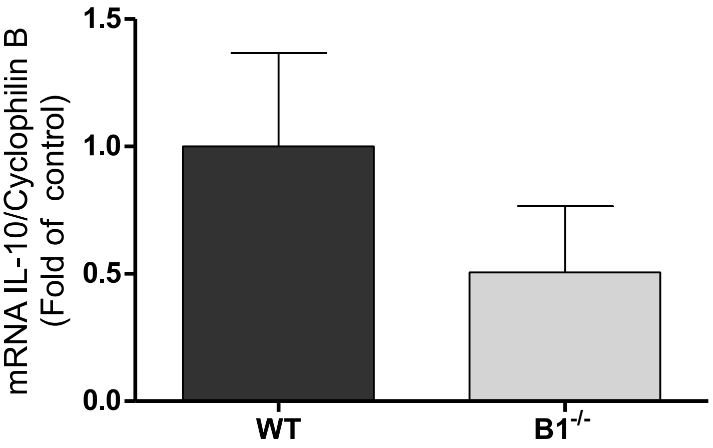

Supplementary Figure S1. Kinin B1 receptor in the host does not alter IL-10 mRNA expression in the lungs during lung melanoma metastasis. Quantitative real-time PCR analysis of IL-10 mRNA expression in B16F10 metastatic lungs induced by the injection of B16F10 cells in the tail vein of wild type (WT) and kinin B1 receptor-knockout mice ($B1^{-/-}$) mice. Data are expressed as fold of change from relative expression of IL-10/Cyclophilin B ±SEM; n=6, p=0.3869.

Supplementary Material – Maria et al, 2016. Sci. Rep.

Supplementary Table

Supplementary Table S.: Oligonucleotide sequences used for the quantitative real-time PCR analysis

Target gene	PCR fragment size (bp)	Forward sequence (5’ → 3’)	Reverse sequence (5’ → 3’)
**IL-6**	280	5’CATCCAGTTGCCTTCTTGGG3’	5’CCAGTTTGGTAGCATCCATC3’
**IL-10**	193	5’GGTTGCCAAGCCTTATCGGAAATGA3’	5’TTCACCTGCTCCACTGCCTTGCT3’
**TNF-α**	140	5’AAGCCTGTAGCCCACGTCGTA3’	5’AGGTACAACCCATCGGCTGG3’
**TGF-β**	94	5’GCAACATGTGGAACTCTACCAG3’	5’CAGCCACTCAGGCGTATCA3’
**IFN-γ**	179	5’CAGCAACAGCAAGGCGAAAAAGG3’	5’AATCTCTTCCCCACCCCGAATCA3’
**VEGF**	77	5’ACTGGACCCTGGCTTTACTG3’	5’TCTGCTCTCCTTCTGTCGTG3’
**Cyclophilin B**	300	5’AAGGACTTCATGATCCAGGG3’	5’TGACATCCTTCAGTGGCTTG3’
